# Adenosine, lidocaine and Mg^2+^ update: teaching old drugs new tricks

**DOI:** 10.3389/fmed.2023.1231759

**Published:** 2023-09-27

**Authors:** Geoffrey P. Dobson, Jodie L. Morris, Hayley L. Letson

**Affiliations:** Heart and Trauma Research Laboratory, College of Medicine and Dentistry, James Cook University, Townsville, QLD, Australia

**Keywords:** adenosine, lidocaine, magnesium, trauma, hemorrhage, inflammation, coagulopathy

## Abstract

If a trauma (or infection) exceeds the body’s evolutionary design limits, a stress response is activated to quickly restore homeostasis. However, when the injury severity score is high, death is often imminent. The goal of this review is to provide an update on the effect of small-volume adenosine, lidocaine and Mg^2+^ (ALM) therapy on increasing survival and blunting secondary injury after non-compressible hemorrhagic shock and other trauma and infective/endotoxemic states. Two standout features of ALM therapy are: (1) resuscitation occurs at permissive hypotensive blood pressures (MAPs 50–60 mmHg), and (2) the drug confers neuroprotection at these low pressures. The therapy appears to reset the body’s baroreflex to produce a high-flow, hypotensive, vasodilatory state with maintained tissue O_2_ delivery. Whole body ALM protection appears to be afforded by NO synthesis-dependent pathways and shifting central nervous system (CNS) control from sympathetic to parasympathetic dominance, resulting in improved cardiovascular function, reduced immune activation and inflammation, correction of coagulopathy, restoration of endothelial glycocalyx, and reduced energy demand and mitochondrial oxidative stress. Recently, independent studies have shown ALM may also be useful for stroke, muscle trauma, and as an adjunct to Resuscitative Endovascular Balloon Occlusion of the Aorta (REBOA). Ongoing studies have further shown ALM may have utility for burn polytrauma, damage control surgery and orthopedic surgery. Lastly, we discuss the clinical applications of ALM fluid therapy for prehospital and military far-forward use for non-compressible hemorrhage and traumatic brain injury (TBI).

## Introduction

We have been developing ALM therapy for hypotensive resuscitation for over 15 years. In 2016 we summarized the developments to that date, which included treatment for myocardial ischemia–reperfusion injury, cardiac arrest, polymicrobial sepsis and hemorrhagic shock (1, 2). The present update focuses on ALM development for hypotensive resuscitation and traumatic brain injury (TBI) with the goal for clinical translation as a small-volume prehospital therapy for civilian and military far-forward use.

## Evolution, design limits and physiological reserve

The living being is an agency of such sort that each disturbing influence induces by itself the calling forth of compensatory activity to neutralize or repair the disturbance.

Léon Fredericq (1885) Quoted from Cannon (3), p. 399

Although Fredericq’s ideas have been lost to the archives for over 130 years, his writings on design agency still resonate in Walter Cannon’s concept of homeostasis, which was formulated around 1926 (3). Cannon acknowledged Fredericq’s ideas and those of Claude Bernard, Eduard Pfluger and Charles Richet, in developing his principles of the steady-state and self-regulation (1, 3). Design agency helps define the limits an organism can tolerate when placed under environmental or physiological stress. If a trauma (or infection) exceeds the body’s evolutionary design limits, a stress response activates the sympathetic nervous system and secondary processes in an attempt to quickly restore whole body homeostasis (4, 5). Multiple factors that influence the body’s ability to restores homeostasis, including the type and severity of injury, prehospital and medical care proficiencies and the patient’s physiological reserve, which itself depends upon age, sex and other genetic and non-genetic influences (6). In addition, if a patient requires emergent surgery, the surgery itself can elicit further traumatic stress from the barrage of damage signals triggered by intervention, despite a successful operation (6, 7). Our working hypothesis to successfully treat an injury (or infection) is to prevent the system from ‘overshooting’ its design limits, and thereby reduce the impact of the primary trauma and secondary injury complications. Before we discuss the development of ALM therapy in treating trauma, we will briefly discuss the importance of the system in this context.

## Trauma: the whole is greater than the sum of its parts

After physiology has taken Humpty Dumpty apart, it is difficult perhaps (even unfashionable) to put him back together again. Consequently, traditional analytical approaches like those in physiology can be positively misleading when applied to problems involving the performance of intact organism.

George Bartholomew (1919–2006) (8), p. 327

Bartholomew’s point is important. Currently, the price we pay for drilling deeper and deeper into life’s inner workings is having less and less information on how the individual parts make up the whole (9). This reductionist way of thinking has crept into the way we problem-solve, diagnose, treat and prevent diseases (10). Reductionism is the general principle that complex phenomena can be explained by conceptually reducing them to a set of simple variables (8). It assumes that understanding the isolated parts, and their structures, have sufficient explanatory power to provide an understanding of the system. As noted by Bartholomew, however, this process can be misleading or incomplete when applied to the intact organism (8). After major trauma or disease, the practice of identifying and treating one clinical defect at a time, and so on down the line, is an example of this way of thinking, which can often lead to what US surgeon William C. Shoemaker considered: “an uncoordinated and sometimes contradictory therapeutic outcome” (11). Lack of more systems-based approaches in medicine and biology may contribute to why there are so few effective drugs translate from animals to humans (12, 13). The key point is that despite an overwhelming amount of mechanistic data being generated from basic scientific research, its relevance to the workings of the whole animal has not kept pace. Reductionism is important in breaking a complex system into its simpler parts, but it does not do away with the system. We have argued elsewhere that the failure to reconstruct the system can be traced back to the molecular revolution of the 20th century, which began in earnest around 1953 after the discovery of DNA (5). Nobel Laureate Sir Francis Crick embodied this position when he wrote “the ultimate aim of the modern movement in biology is to explain all biology in terms of physics and chemistry” (14). A more detailed historical discussion of the subject of reductionism, and its limitations, can be found in our recent review (5).

## Early work on alm: cardioplegic arrest to resuscitation

There are three stages of scientific discovery: first people deny it is true; then they deny it is important; finally, they credit the wrong person.

Credited to Alexander von Humboldt (1769–1859) (15)

In 1998, GPD asked if it was possible to make the human heart operate more like the heart of a natural hibernator to improve its protection during cardiac surgery (1). For over 60 years, cardioplegia have been based on high potassium (K^+^) solutions to depolarize the heart from −84 to −50 mV, and thereby induce diastolic arrest (16). High K^+^ can induce ischemia during arrest and reperfusion injury after reanimation (16). An alternative idea to arrest the heart more naturally at or near its resting membrane potential was to use: (1) adenosine (A) to inhibit the sinoatrial (SA) node and reduce action potential (AP) duration via the adenosine A1 receptor-linked opening of K_ATP_ channels; (2) lidocaine (L) to reduce AP amplitude by blocking voltage-dependent Na^+^ fast channels, and (3) magnesium (M) to stabilize the cardiac membrane and protect against reperfusion arrhythmias via its ability to raise the threshold for fibrillation (16). The ALM Zero K^+^ cardioplegia idea was subsequently translated from the isolated rat heart to human cardiac surgery (17, 18) and supported by two prospective, randomized, clinical trials in high- and low-risk patients, which showed that ALM cardioplegia was superior to Buckberg high K^+^ solution (17, 18).

The second ALM idea came after observing the rapid reanimation of the human heart following cardiac surgery when 10-times lower concentrations of ALM were infused into the heart for 2–5 min (16). Could this lower dose ALM resuscitate and protect the heart after major trauma? Over the past 12 years, we have shown that small-volume low dose ALM IV infusion protects the heart and whole body against cardiac arrest, regional myocardial ischemia, hemorrhagic shock (1, 2), polymicrobial sepsis (19), endotoxemia (20), TBI (21) and the trauma of major surgery (22). Importantly, the individual actives, A, L or M failed to confer these benefits; it is only the combination that resuscitates and confers whole body protection (1, 23). In our earliest studies, the vehicle was 7.5% NaCl to assist in raising mean arterial pressure (MAP) in shock states and possibly reduce brain edema and intra cranial pressure after TBI. This was later changed to 3% NaCl, which is approved by the FDA, and reportedly reduces oxidative stress (24). Below, we provide a brief update on ALM therapy’s ability to improve central nervous system (CNS) and cardiovascular function, protect the endothelium glycocalyx, and confer mitochondrial metabolic support after different trauma states from studies conducted in our laboratory, as well as others around the world. We will further argue that ALM is emerging as a systems-acting therapy that has wide applications by reducing the progression of secondary injury after trauma, surgery or infection. Lastly, we will discuss possible mechanisms underlying ALM’s short- and long-term benefits which appear to involve NO synthesis pathways (25).

## Addressing a major gap in resuscitation: permissive hypotension

The concept of “permissive hypotension” refers to managing trauma patients by restricting the amount of resuscitation fluid and maintaining blood pressure in the lowest than normal range if there is continuing bleeding during the acute period of injury.

Kudo et al. (26), p. 1

**Figure 1:**
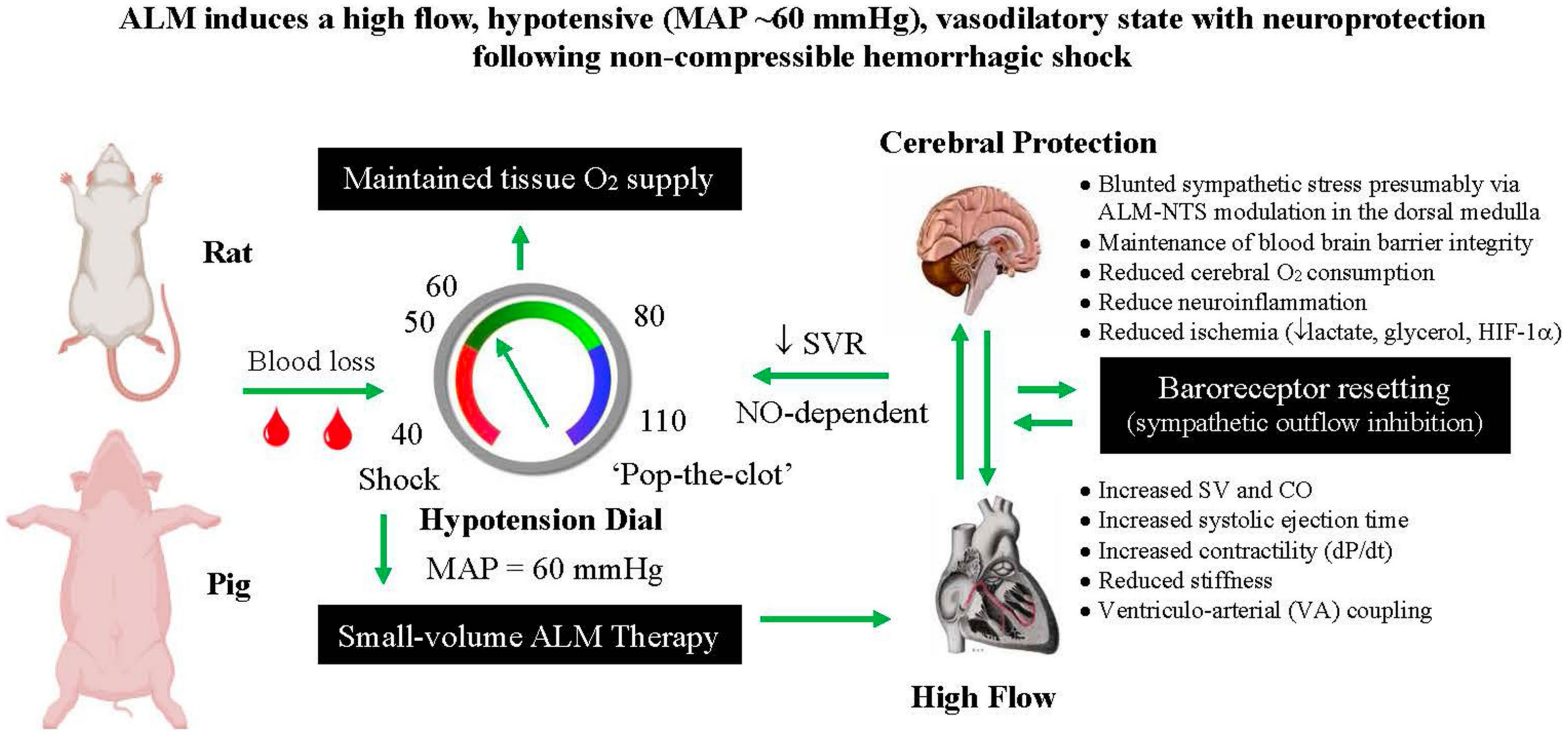
Schematic of ALM’s ability to resuscitate at permissive hypotensive mean arterial pressures (MAPs). The proposed resuscitation sequence is as follows: Hemorrhagic shock (MAP 30–40 mmHg) → Small-volume bolus ALM → increased cardiac output (CO) and stroke volume (SV) → nitric oxide (NO)-dependent MAP increase → pulsatile stretch activates baroreceptors → afferent nerve signals to brain via glossopharyngeal and aortic nerves → synapses with the nucleus tractus solitarius (NTS) in the medulla → sympathetic outflow to vasodilate arterial vasculature → systemic vascular resistance (SVR) decreases → feedback resetting of MAP via baroreceptor resetting, cardiovascular (ventriculo-arterial) VA coupling → preserved tissue O_2_ delivery → Increased SURVIVAL. ALM, adenosine, lidocaine and magnesium; HIF-1α, hypoxia inducible factor 1-alpha.

Most trauma deaths are secondary to CNS injury, non-compressible bleeding, airway insufficiency, and multiple organ failure (5). Hemorrhage contributes to 33–56% of pre hospital deaths (27), which increases to 90% in far-forward military environments (28). The first goal of first responders is to stop the bleeding, raise MAP if the patient is in shock, prevent rebleeding, and stabilize the casualty. Currently, there is no fluid therapy to effectively resuscitate and stabilize the patient. Key features of ALM’s anti-shock therapy in rats and pigs is its capability to resuscitate the heart, provide neuroprotection and reduce internal bleeding *at hypotensive MAPs* (50–60 mmHg) after hemorrhagic shock (Figure 1) (1, 2, 29–31). A concern with past hypotensive therapies is brain ischemia and functional loss from insufficient cerebral blood flow (CBF) (32). Neuronal dysfunction occurs when cerebral perfusion pressure (CPP) falls to ~40% of normal, and irreversible damage occurs at 20% CPP if sustained for >1 h (32). Hypotensive resuscitation is especially contraindicated in patients with TBI for the same reasons (26).

To our knowledge, ALM is the first fluid therapy that resuscitates into the permissive hypotension range. In the pig model of non-compressible hemorrhagic shock, despite a significant decrease in CPP (~25%), ALM-treated animals has significantly reduced cerebral oxygen consumption (28%), reduced brain glycerol (60%) and lactate (47%), and lowered relative gene expression of hypoxia-inducible factor-1α (HIF-1α) (38%) compared with saline controls (31). ALM therapy appears to solve four problems in damage control resuscitation; (1) MAP is raised to hypotensive MAPs (~60 mmHg) using small volumes (1–4 mL/kg), (2) non-compressible blood loss is reduced (presumably via ALM coagulopathy correction), (3) rebleeding or ‘popping the clot’ is minimized, and (4) cerebral protection is afforded at these lower pressures (Figure 1). ALM-induced neuroprotection also occurs after moderate TBI with 3-fold increases in cortical blood flow, lower cortical and systemic inflammation (up to 70% fall) and significantly reduced neuronal injury compared to saline controls (see below) (21).

## The CNS stress response: first pillar to control sympathetic hyperdrive

Shock is the “a rude unhinging of the machinery of life.”

Samuel Gross (1862) Quoted from (33), p. 437

The importance of CNS control of traumatic injury cannot be overstated (34). The extent of CNS ‘unhinging’ depends on stress signals being received from damaged peripheral nerves and circulating damage-associated molecular patterns (DAMPs), cytokines and chemokines from the site of injury, which can move across the blood brain barrier (BBB) and alter CNS autoregulatory functions (4, 35). If this early stress response can be suppressed, it is possible that the secondary injury processes driven by uncontrolled sympathetic discharge may be attenuated (36).

**Figure 2:**
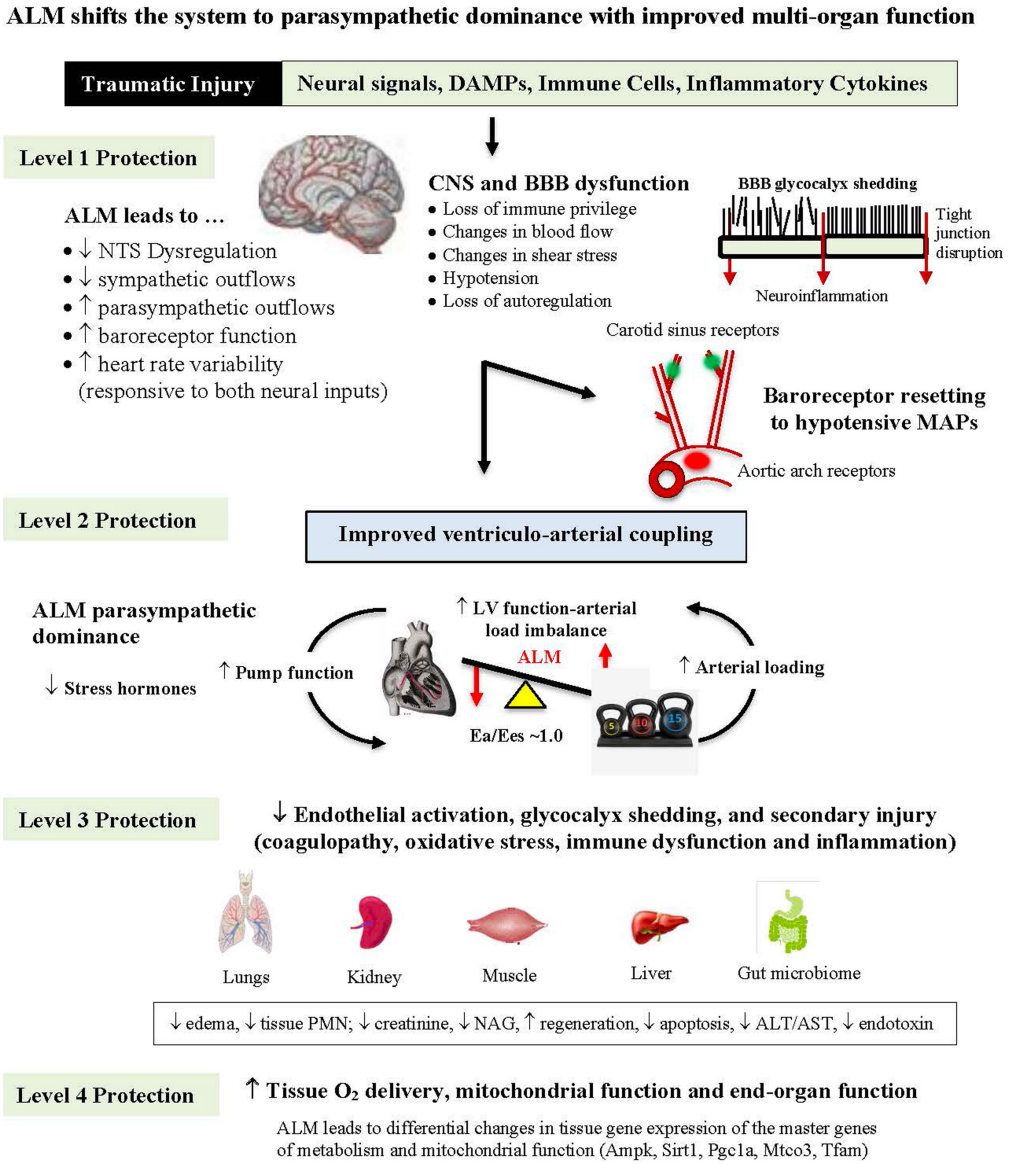
Diagram showing how ALM therapy blunts the sympathetic hyperdrive stress response following hemorrhagic shock with multiorgan protection from brain to mitochondria. ALM reduces nucleus tractus solitarius (NTS) sympathetic outflows and possibly protects the blood brain barrier (BBB) from losing its immune privilege status over homeostatic control with improved ventriculo-arterial (VA) coupling and tissue O_2_ delivery (see text). ALM, adenosine, lidocaine and magnesium; DAMP, damage-associated molecular pattern; CNS, central nervous system; MAP, mean arterial pressure; LV, left ventricle; Ea, arterial elastance; Ees, end-systolic elastance; PMN, polymorphonuclear leukocyte; NAG, N-acetyl-beta-glucosaminidase; ALT, alanine aminotransferase; AST, aspartate aminotransferase; Ampk, AMP-activated protein kinase; Sirt1, sirtuin 1; Pgc1a, peroxisome proliferator-activated receptor gamma coactivator 1-alpha; Mtco3, cytochrome c oxidase subunit III; Tfam, transcription factor A, mitochondrial.

Five lines of evidence from animal models suggest ALM therapy blunts the CNS stress response [Figure 2; (36)]: (1) ALM infusion led to a 30-fold increase in parasympathetic/sympathetic receptor expression ratio in the heart reflecting a switch from sympathetic to parasympathetic dominance responsible for the improved cardiac function and reduced systemic inflammation (36); (2) its ability to protect the cerebral cortex in the pig model of non-compressible hemorrhagic shock by significantly lowering ischemic biomarkers (see above) (31); (3) from an independent study of Wang and colleagues showing that ALM therapy led to a significant reduction of neuronal injury markers neuron-specific enolase (NSE), S100 calcium binding protein (S100B) and matrix metalloproteinase (MMP-9) in a rat ischemic stroke model (37), (4) its ability to significantly reduce release of the DAMP high mobility group box protein 1 (HMGB1) and brain injury markers (NSE, syndecan-1), following moderate TBI in a rat model (21); and (5) its ability to prevent TBI-induced neurogenic cardiac failure and provide multiorgan protection [Figure 2; (38)]. ALM-CNS modulation in different trauma models by us, and others, support further translational studies to human safety trials.

## Cardiovascular support: second pillar to improve trauma outcomes

Little evidence exists to guide the use of crystalloid in the setting of prehospital resuscitation after injury, and a universal approach to prehospital resuscitation does not exist.

Weykamp et al. (39), p. 28

Concern over crystalloid (ab)use use has a long history. In 1911, Evans wrote the following in JAMA: “One cannot fail to be impressed with the danger of…the utter recklessness with which salt solution is frequently prescribed, particularly in the postoperative period…” (40). We argue the same applies today. The main goal of fluid resuscitation is to support cardiovascular function to deliver adequate oxygen to the tissues of the body. However, current therapies fail to increase cardiac output (CO) in up to 50% of trauma patients (41), which is particularly problematic in hemorrhaging patients with aggravation of coagulopathy, inflammation and tissue hypoperfusion (42). Smaller resuscitation fluid volumes are now recommended (200–500 mL) (43), however, this does not assist non-responders. In addition, an unappreciated fact is that around one-third of major trauma patients have abnormal echocardiographic ventricular wall motion, lower CO, and increased plasma troponin 1, despite inotrope and vasopressor support (42). Restoring cardiac function and tissue O_2_ delivery following hemorrhagic shock is a major gap in prehospital trauma care.

In rat models of up to 60% blood loss and pig models of 75% blood loss, improved cardiovascular function during small-volume ALM resuscitation was associated with significant increases in stroke volume (SV), CO and systolic ejection time, which was associated with improved tissue O_2_ delivery and reduced blood lactate (1, 2, 44, 45). A standout feature of ALM resuscitation is a fall in systemic vascular resistance (SVR) and therefore afterload on the left ventricle compared to saline controls [Figures 1, 2; (31)]. A more compliant system with higher arterial elastance may be the result of the following: (1) ALM’s ability to reduce sympathetic outflows and oscillations from the CNS to the periphery, and (2) ALM’s effect to reduce arteriolar tone and opening of vascular beds. A reduced arterial tone is supported by recent studies showing ALM solution relaxes rat thoracic aortic rings and guinea pig mesenteric arteries, and this vasodilatory effect of ALM may contribute to lowering SVR (46). A lower SVR after ALM treatment has also been shown in pigs during lipopolysaccharide (LPS)-induced endotoxemia (20). ALM therapy decreased SVR by 60% compared to 20% in saline controls, and surprisingly maintained CO and O_2_ delivery at a hypotensive MAPs of ~47 mmHg over 4 h (20). A MAP of 47 mmHg typically is considered the lower limit of vascular autoregulation and incompatible with life (47). This ALM hypotensive state during hemorrhage and endotoxemia is in direct contrast to systemic hypotension that develops from failure of cardiovascular function after major trauma.

We termed this new ALM-induced set-point as: “a high-flow, hypotensive, vasodilatory state with improved O_2_ delivery and cerebral protection” (31). O_2_ delivery was maintained in part by ALM’s ability to optimize ventriculo-arterial (VA) coupling [Figure 2; (5, 17, 20, 45)]. VA coupling is the ratio of arterial elastance (Ea) to left-ventricular (LV) elastance (Ees) and reflects the efficiency of the heart to eject blood and the ability of the arterial system to receive it (48). When the Ees/Ea ratio is close to unity, the efficiency of transfer is considered optimal. If the ratio is excessively low or high, the heart as a pump and the vascular load become uncoupled and tissue O_2_ perfusion is compromised (17, 45, 48). In the case of CNS dysregulation, if the proximal arteries become less compliant and afterload increases or if the heart cannot relax optimally, the heart’s ability to eject blood is reduced with subsequent loss of SV, CO and tissue O_2_ delivery (5). *The clinical advantage of VA coupling over ejection fraction (EF) is that it provides both LV function* and *arterial load properties* (48).

From a system’s perspective, we propose that ALM maintains VA coupling by resetting the CNS-baroreflex, dilating the arterial system (indicated by a fall in SVR, see above), restoring LV contractility and improving LV relaxation (Figures 1, 2). Relaxing the myocardium and increasing systolic ejection time appears to be a unique property of ALM therapy and occurs during reanimation of the heart following cardiac surgery (17, 18). University of Verona Hospital cardiac surgeons, who conducted two prospective randomized ALM cardioplegia trials in high and low-risk patients, also reported restoration of ALM-linked VA coupling, increased contractility and relaxation (17, 18). More studies are required to examine the underlying molecular mechanisms including Ca^2+^ handling during the cardiac cycle and arterial compliance, as well as a role of nitric oxide (NO) (see below) and mitochondrial integrity. Future studies in trauma care will hopefully provide more context and utility of VA coupling (Ees/Ea ratio) for goal-directed resuscitation to potentially predict and improve patient outcomes (5). In summary, ALM’s resuscitative effect to produce a high flow, hypotensive, vasodilatory state to maintain tissue O_2_ delivery appears to be unique compared to other fluid therapies, and provides the preclinical support to the next steps of human translation.

## ALM resuscitation, survival and stabilization is nitric oxide (NO)-dependent

The amplification of vascular smooth muscle cell sensitivity to NO is suggested to be a new mechanism in baroreflex physiology, which can promote interactions between the sympathetic nervous system and NO function.

Gmitrov (49), p. 143

**Figure 3:**
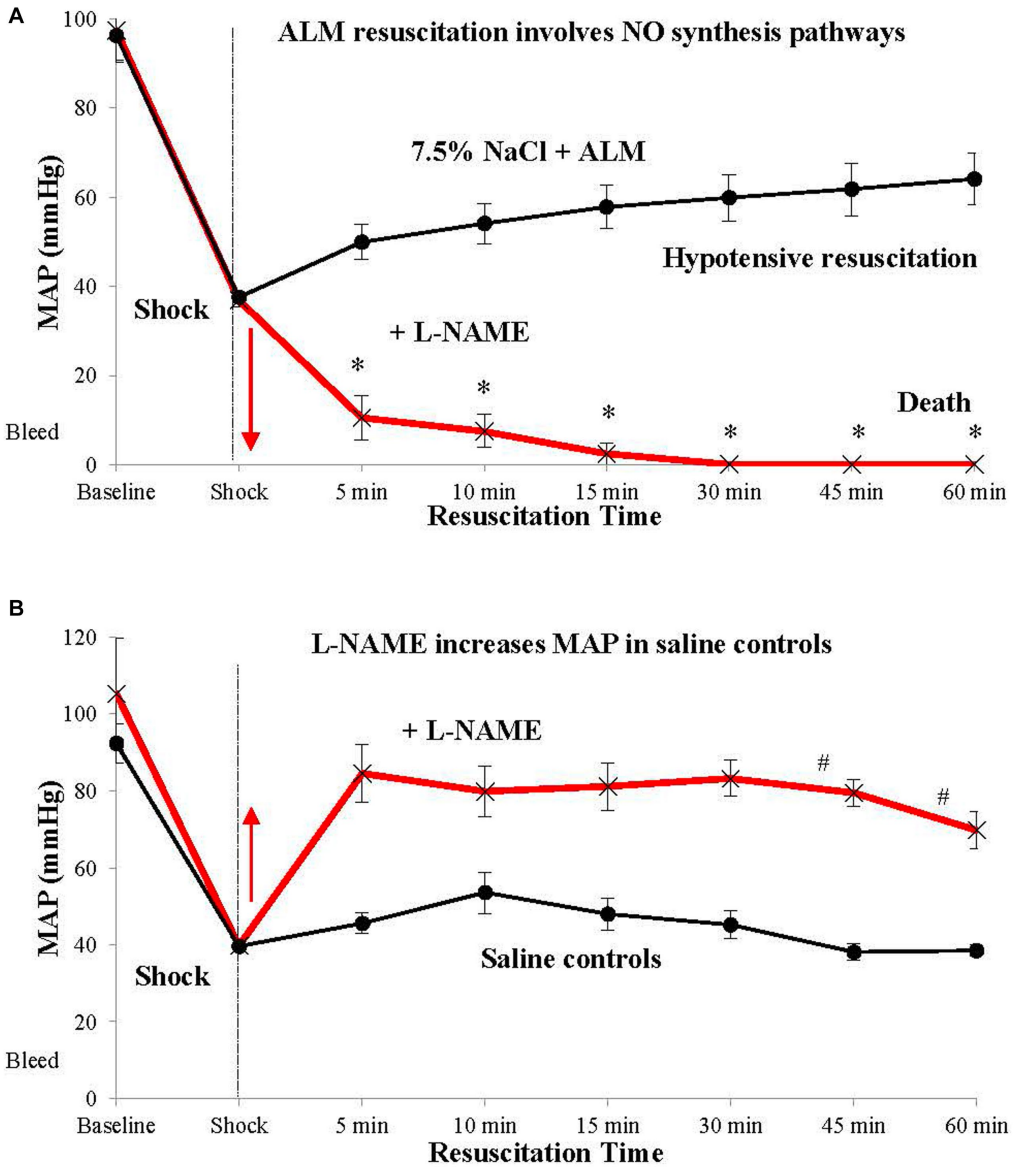
ALM resuscitation, stabilization and survival involves nitric oxide (NO) synthesis pathways. ALM therapy in the presence of L-NAME fails to resuscitate after hemorrhagic shock **(A)**. In contrast, saline in the presence of L-NAME resuscitates MAP to ~80 mmHg, likely due to systemic vasoconstriction. **(B)** Mechanistic studies involving specific nitric oxide synthase (NOS) inhibitors showed that resuscitation failure with ALM + L-NAME was not due to neuronal NOS, but most likely involved endothelial NO-producing (eNOS) pathways (25). ALM, adenosine, lidocaine and magnesium; L-NAME, L-NG-Nitro arginine methyl ester; MAP, mean arterial pressure.

Another curious finding was that ALM’s ability to resuscitate MAP is abolished in the presence of the nonselective NO synthase (NOS) inhibitor L-N^G^-Nitro arginine methyl ester (L-NAME) [Figure 3; (25)]. It is widely known that L-NAME increases MAP following hemorrhagic shock via its cardiac and arteriolar vasoconstrictive effects (50). We confirmed this MAP-raising effect in our saline controls in a rat hemorrhagic shock model where MAP increased from ~30 to ~60 mmHg following bolus administration of 7.5% NaCl + (L-NAME) [Figure 3; (25)]. *The inability of ALM to resuscitate MAP in the presence of L-NAME indicates activation of one or more NO-producing pathways for its normal operation* (25). Two likely candidates for ALM-NO interactions are possible. First, is the effect of L-NAME on the medulla’s NTS, which is known to express both endothelial and neuronal forms of NOS (51), and controls the sympathetic and parasympathetic outputs to the heart, which ALM appears to modulate (52, 53). Second, NO receptors are implicated in systemic vascular compliance, which would impact on ALM’s ability to lower SVR and resuscitate MAP (discussed above).

With respect to the first possible mechanisms, modulation of adenosine A1 receptors within the NTS, and possibly lidocaine’s actions, are known to control cardiovascular function *in vivo* (54). Activation of adenosine receptors and NO pathways have been shown to differentially inhibit or reset the baroreflex control of MAP, HR, and renal sympathetic nerve activity (54, 55). Bilateral microinjections of lidocaine into the NTS have also been shown to increase MAP in alpha-chloralose-anesthetized control rats (56). Wang and colleagues further showed that the tonic blockade of cardiac sympathetic afferent reflex by epicardial lidocaine in chronic heart failure experiments can reduce the activity of NTS chemoreceptive neurons, and alter sympathetic outflows to the heart, and possibly other organs (57). Thus, ALM’s ability to resuscitate MAP following hemorrhagic shock, and other trauma states, appears to involve crosstalk between NO within the NTS, and resetting of the baroreflex and cardiovascular coupling (25).

## ALM restores endothelial-glycocalyx: third pillar of cellular protection

Combined with direct in vivo measurements showing full restoration of glycocalyx thickness and recovery of vascular hyperpermeability, the data suggests that ALM salutary effects may be linked to its beneficial actions at the level of microvascular glycocalyx.

Torres Filho et al. (58)

Trauma-associated injury to the endothelial-glycocalyx is termed Endotheliopathy of Trauma (EoT) (59). EoT is characterized by endothelial activation, heightened vasoreactivity, fluid shifts, leakiness, leukocyte adhesion, inflammation, coagulopathy and mitochondrial dysfunction (59). Activation and damage can lead to glycocalyx shedding and the release of endothelial-bound thrombomodulin, tight junction proteins, syndecan-1, heparan sulfate, hyaluronic acid, and other proteoglycans and glycoproteins, into the circulation (60). These injury markers also indicate widespread tissue damage, including damage to loss of BBB integrity (61) and amplification of the CNS stress response (4, 5).

Independent studies by Dubick and colleagues at the US Army Institute of Surgical Research have reported that ALM therapy almost completely reversed endothelial glycocalyx damage (97%) after severe hemorrhagic shock (58). We have also reported similar reductions in endothelial injury marker syndecan-1 after hemorrhagic shock and TBI (21). Restoration of the endothelial-glycocalyx is consistent with ALM’s rapid correction of coagulopathy and blunting of systemic inflammation (see below) (4, 21, 23, 36, 62), and resetting of the CNS-cardiovascular and O_2_ delivery systems to tissue mitochondria (4, 5). A healthy endothelial-glycocalyx is essential for rapid recovery after major trauma. The vastness of endothelium is often overlooked; it covers a surface area (SA) of 3,000–7,000 m^2^ (63), and *if* the glycocalyx “fuzz” is included, we have estimated the SA increases around 10-fold (46,000 m^2^ or ~ 200 tennis courts or eight USA football fields) (4). Further work is required to examine the mechanisms underlying ALM’s ability to restore and protect the endothelial-glycocalyx, and its relation to secondary injury reduction (coagulopathy, inflammation, immune dysfunction and metabolic stress) (discussed below).

## ALM reduces inflammation and corrects coagulopathy

Increasing evidence points to an extensive cross-talk between these two systems, whereby inflammation leads to activation of coagulation, and coagulation also considerably affects inflammatory activity.

Levi and Van der Pol (64), S26

**Figure 4:**
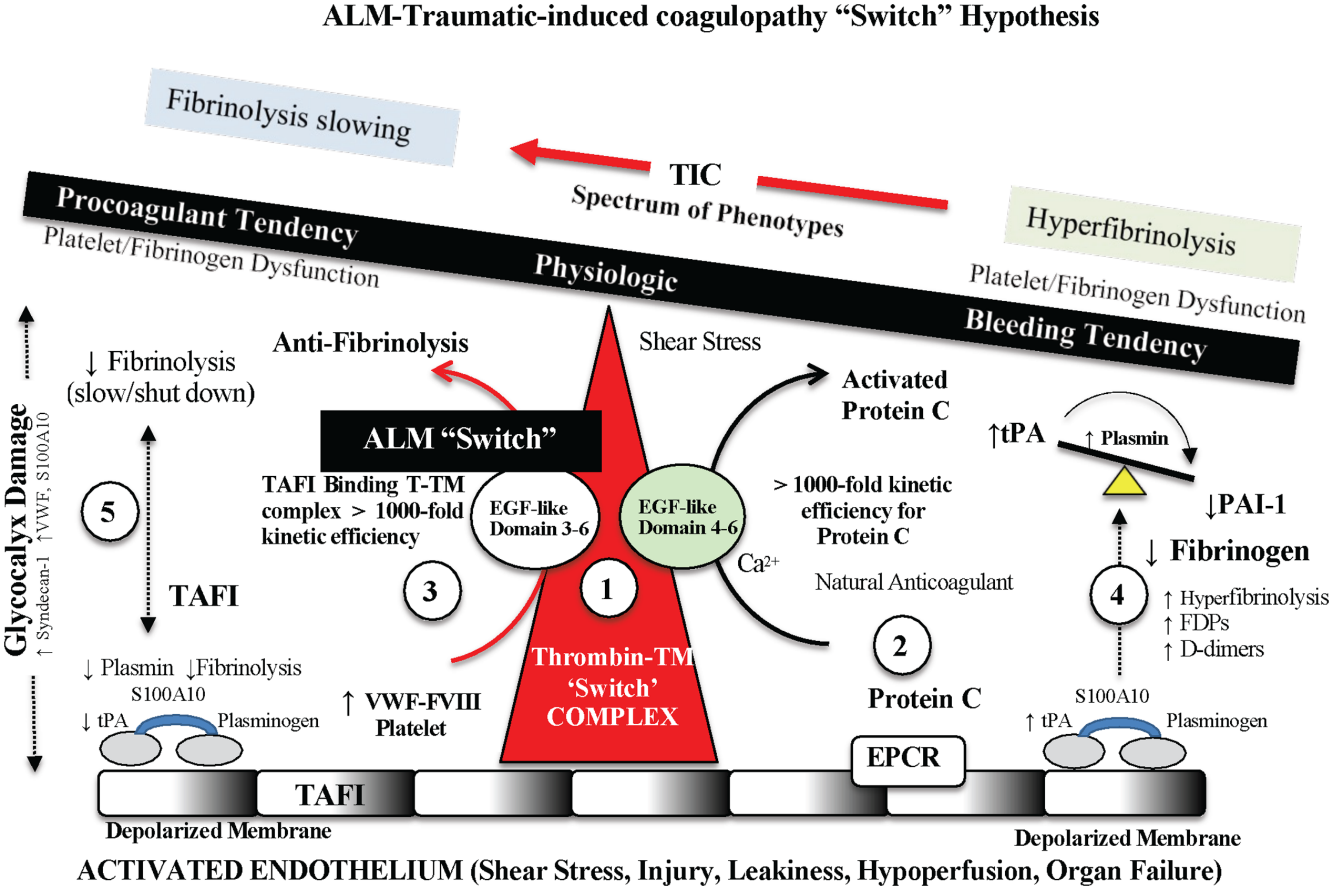
Possible endothelial-glycocalyx mechanism for rapid TIC correction with ALM therapy. ALM acts like a “switch” at the thrombomodulin (TM)-thrombin–annexin II complex and corrects trauma-induced coagulopathy (TIC) by shifting the complex’s thrombin substrate specificity from the protein C pathway to the thrombin activatable fibrinolysis inhibitor (TAFI) pathway, and therefore prevents hyperfibrinolysis. The site we propose is ALM-linked preferential binding of thrombin on epidermal growth factor (EGF)-like domains 3–4 located on the thrombin-TM luminal stalk, and possibly involving annexin 2 on the endothelial surface (65). Anticoagulant protein C interacts with domains 4–6, and procoagulant TAFI binds to domains 3–4 (65). ALM, adenosine, lidocaine and magnesium; TPA, tissue plasminogen activator; PAI-1, plasminogen activator inhibitor-1; VWF, Von Willebrand factor; S100A10, S100 calcium binding protein A10; FVIII, Factor VIII; EPCR, endothelial protein C receptor; FDP, fibrin degradation product.

An outstanding feature of ALM is that it significantly reduces local and systemic inflammation and corrects trauma-induced coagulopathy (TIC) and hyperfibrinolysis after hemorrhage, TBI, sepsis, endotoxemia, burns and major surgery in rat and pig models (2, 30, 62, 65). The rapid correction (within 5 min) and anti-fibrinolytic properties in ALM-treated animals compared to saline controls is the likely reason for the significant 60% reduction in non-compressible bleeding after hemorrhagic shock (62). *Small-volume ALM acts like an ‘internal’ pharmacological tourniquet by rapidly correcting TIC.* The data further suggests that TIC was not consumptive because clotting factors, post-shock platelets, and coagulation pathways were all present to be corrected so quickly (62, 65). Our current working hypothesis is that ALM acts like a “switch” at the thrombomodulin (TM)-thrombin–annexin II complex located on the endothelium and corrects TIC by shifting the complex’s thrombin substrate specificity from the protein C pathway to the TAFI pathway (62, 65). The site we propose that leads to the TAFI direction is preferential binding of thrombin on EGF-like domains 3–4 located on the thrombin-TM luminal stalk, and possibly involving annexin 2 on the endothelial surface (65). Anticoagulant protein C interacts with domains 4–6, and procoagulant TAFI binds to domains 3–4 [(65); Figure 4].

**Figure 5:**
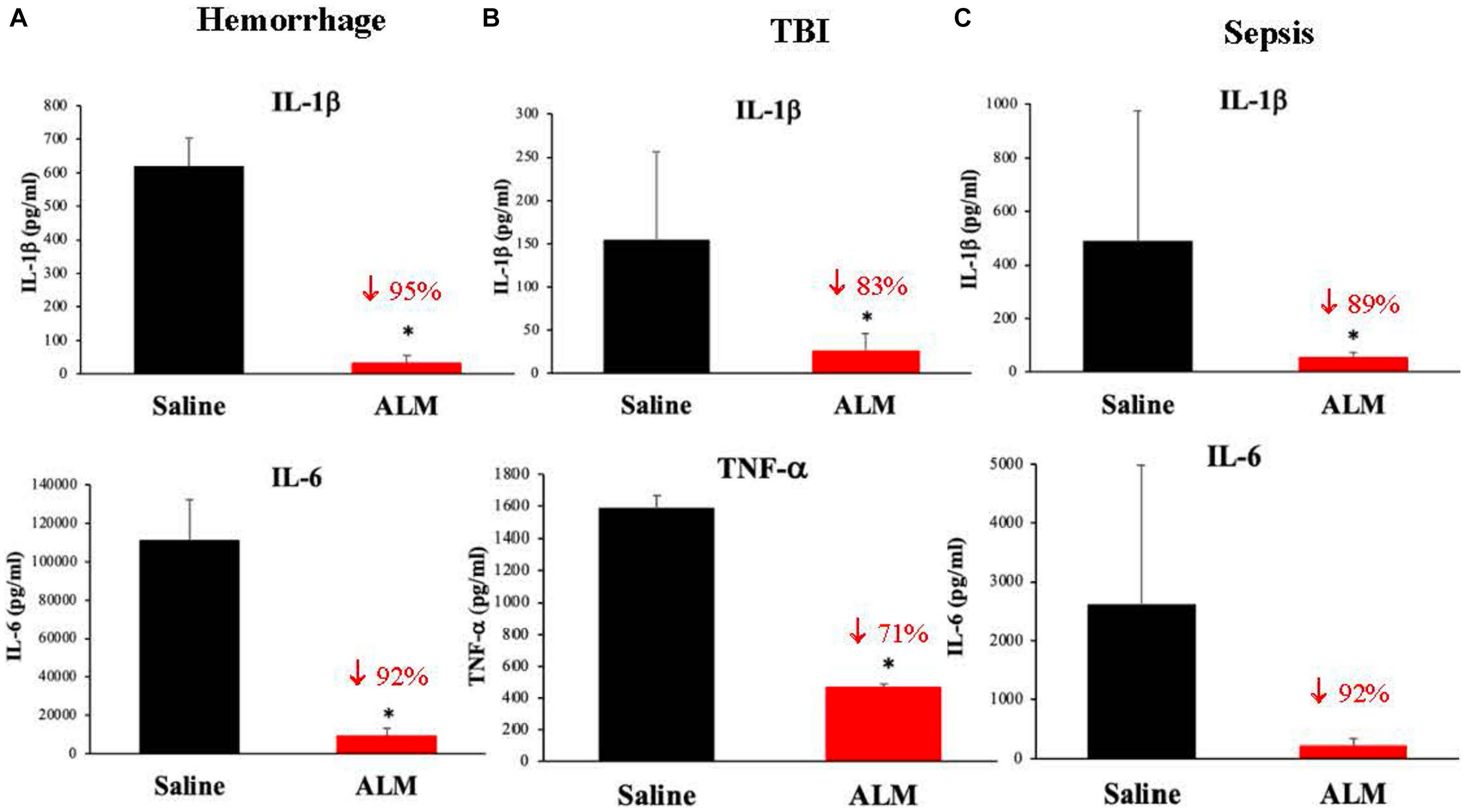
ALM’s effect to dramatically inhibit secondary injury from inflammation following hemorrhagic shock **(A)** (30), TBI **(B)** (21) and polymicrobial sepsis **(C)** (19). Interleukin (IL)-1β is a key immunoregulatory and proinflammatory cytokine that is produced by the NLR family pyrin domain containing 3 (NLRP3) inflammasome, a caspase-1 activating molecular platform that responds to damage-associated molecular patterns (DAMPs) and pathogen-associated molecular patterns (PAMPs) (4). Tumor necrosis factor (TNF)*-*α and IL-6 are also important transcriptional regulators of NLRP3 inflammasome components which regulate downstream inflammatory processes. IL-6 is particularly important in amplifying immune responses upon pathogen infection. ALM, adenosine, lidocaine and magnesium; TBI, traumatic brain injury. *p < 0.05.

The anti-inflammatory effect of ALM is equally robust as TIC/fibrinolytic correction (20, 21, 30, 31), adding further evidence of ALM’s ability to restore endothelial and mitochondrial function. ALM’s anti-inflammatory and immunomodulatory effects includes 80% falls of IL-1β, TnFα and IL-6 after hemorrhage, TBI and sepsis [(20, 21, 30, 31); Figure 5]. IL-1β is a key signature cytokine and activates the inflammasome and downstream inflammatory processes (4). Like the rapid correction of TIC, ALM appears to act in the first minutes to hours post-injury to *rebalance the system with timely resolution of systemic inflammation and immune dysregulation* (4, 5).

## Mitochondrial oxidative phosphorylation: fourth pillar of cellular protection

The strategic positioning and abundance of mitochondria ensure a highly efficient localized ATP delivery system to support contraction, metabolism, and ion homeostasis. However, mitochondria are also important regulators of cell death, responding to a wide variety of stress signals.

Gustafsson and Gottlieb (66)

Three lines of evidence support that ALM improves mitochondrial function: (1) increased cardiac output and maintenance of tissue blood flow after different trauma and endotoxemia states (20, 29–31), (2) reduced systemic blood lactate compared to saline controls (2, 21, 31), and (3) changes in the differential expression of key metabolic genes in central organs heart and brain favoring mitochondrial metabolism (67). These characteristics help to define the 3-day ALM survival phenotype in the rat model of non-compressible hemorrhage, and includes upregulation of Ampk, Sirt1, Pgc1a, and Mtco3 in heart and brain compared to peripheral tissues (67). Amp-k is an upstream molecular sensor that responds to metabolic stress (68), and Sirt-1 expression helps to match cellular energy supply to demand (69). Pgc1a is another sensor that can lead to increased ATP production via activation of mitochondrial respiratory genes, including cytochrome c and β-ATP synthase (70). Lastly, Mtco3 gene encodes for one of three subunits of Complex IV, the irreversible and terminal step of the mitochondrial electron transport chain (67). We reported that Mtco3 expression significantly increased 19-fold in heart and was 2.3-fold higher in brain relative to saline controls up to 3 days following hemorrhagic shock (67). In addition, we have shown Tfam, a gene involved in mitochondrial biogenesis was significantly increased in heart and brain in ALM-treated animals 3 days following trauma (5, 71). Thus, ALM therapy appears to transcriptionally reprogram the heart and brain as part of a survival phenotype compared to controls following trauma.

A summary of how ALM systems-acting therapy increases survival from the CNS to mitochondria following trauma is outlined in Table 1. A challenge for the future is to understand how ALM ‘switches’ the genomic and proteomic regulatory networks at the cellular and mitochondrial level from an injury phenotype to a survival phenotype. Further, how does ALM provide long-term protection when the half-lives of individual components A, L and M are only seconds to a few hours (1, 67)?

**Table 1:** ALM survival phenotype.

Improved CNS function
Improved cardiovascular function
Improved blood flow to brain, gut and kidney
Improved tissue oxygenation
Endothelial-glycocalyx protection
Reduced systemic inflammation
Corrected coagulopathy
Improved platelet function
Reduced immune activation
Differential expression of master genes of metabolism in central and periphery
Upregulation of mitochondrial bioenergetic capacity in heart and brain
Reduced mitochondrial oxidative stress
Reduced ischemic injury

## ALM survival is contradicted in presence of analgesic buprenorphine

Not all trauma research using ALM has shown survival properties. In contrast to our work over a decade in three different rat and pig traumatic hemorrhage models (two pressure-controlled, volume-controlled and one non-compressible hemorrhage), the San Antonio Naval Medical Research Unit (NAMRU) reported ALM therapy was suboptimal to standard TCCC practice (72). In a pig model of pressure-controlled bleeding, How and colleagues reported that ALM was inferior to Hextend (72), which itself has been shown to be suboptimal (2). How can our findings be reconciled with theirs? *The short answer appears to be their use of buprenorphine as an analgesic.* Buprenorphine is an opioid and has a number of adverse events including cardiac and respiratory depression, coagulopathy and immunomodulatory effects (73).

On the basis of How’s findings, and our own sepsis studies, we subsequently conducted a number of ALM studies comparing buprenorphine with the non-steroidal, anti-inflammatory drug (NSAID) Carprieve® (carprofen). We found similar results to How et al., (74, 75), including buprenorphine-linked reduced survival and coagulopathy (75). This represented an important advance irrespective of whether ALM is present or not, because buprenorphine is widely used in preclinical animal studies. Based on our studies and the literature findings of others in sepsis and burn models, we have argued that buprenorphine analgesic should not be used in trauma research (74–76). The conflicting results on ALM’s resuscitation ability reinforces the clinical importance of performing dose safety studies in small and large animals prior to human translation. In 2020, we successfully completed the first ALM toxicology study according to ICH guidelines and showed no adverse effects in rats or pigs over a range of ALM concentrations up to eight times those expected to be used clinically or in the field. Fortunately, buprenorphine is not used widely as an analgesic in trauma centers around the world or by combat medics in the field or for damage control surgery. The most commonly used analgesic in prehospital civilian and military emergency medicine is ketamine (77, 78), which has no contraindications with ALM as reported in our pig studies (31).

## Potential military and civilian applications: from pre-clinical models to humans

The tragedies of life are largely arterial.

Sir William Osler (1908) Quoted from Criado (79)

In civilian environments, up to 50% of trauma deaths involve hemorrhage (80), and increases to ~90% in forward military environments, where ~25% are potentially preventable (28, 81). A recent retrospective study of UK deaths in Afghanistan found that two-thirds of deaths occurred within 10 min of injury (81). For this reason, austere military and rural and remote environments are particularly challenging. Our goal is to translate ALM therapy for resuscitation and casualty stabilization in these prehospital environments, including mass casualty and terrorist events.

Application of ALM during and following Resuscitative Endovascular Balloon Occlusion of the Aorta (REBOA) to reduce ischemia, coagulopathy and inflammation, is another emerging area of drug development being investigated by different military surgical teams at the Madigan Army Medical Center (82, 83) and San Antonio Military Medical Center (84). In a pig model of 20% controlled hemorrhage, followed by 30 min of supraceliac balloon occlusion, Conner and colleagues showed that a pre-REBOA bolus of ALM followed by 4 h infusion reduced ischemia and significantly improved lactate, base deficit, and pH in the first hour following systemic reperfusion. A year later, the same group showed after 45 min of supraceliac balloon occlusion that ALM therapy led to a significant reduction in CO, blunting of systemic inflammation (IL-12, IL-10, IL-4) and reduction of liver injury (83). The San Antonio group used a different approach as part of our Naval Medical Research Unit (NAMRU) collaboration with the group. They examined ALM’s reversible cardioplegia properties to arrest the heart and buy time before performing REBOA to stop the bleeding (84). In an uncontrolled hemorrhage pig model, induced via a 1.5-cm right femoral arteriotomy, Stigall and colleagues showed that ALM-induced cardiac arrest followed 7 min later by REBOA inflation significantly decrease the amount of blood loss by 27% at initial presentation, without compromising survival (84). These are early days. However, the results appear to be promising and survival outcomes may be improved with different timing and optimization of ALM doses (5).

Other potential clinical applications of ALM include TBI, burns, improved healing following injury and trauma of surgery. The TBI preclinical data discussed above was from animals that were anesthetized and mechanically ventilated for 5 h with no cognitive testing (21). Further studies are required in clinically relevant models of mild-to-severe TBI to examine the effect of ALM on blunting secondary injury with conscious monitoring over at least 30 days. With respect to injury and the trauma of surgery, we have shown ALM significantly reduces intraarticular fibrosis compared to saline controls in a rat model of total knee arthroplasty (↓TGF-β1, α-SMA, FGF1, PDGFA, *p* < 0.05, *n* = 6) and it improved range of motion by 2-fold compared to saline controls over the 28-day study period (22). We further showed that ALM therapy reduced relative gene expression of the pro-inflammatory cytokine NF-κB by 66% (*p* = 0.034) and MMP-13 gene expression by 50% in cartilage at day 28, with lesions visualized histologically (22). Our overall goal is to protect the knee joint (and other musculoskeletal tissue) from the trauma of surgery and reduce time to heal and later-life complications. Our most recent work shows that ALM appears to have sex-specific joint healing properties following anterior cruciate ligament (ACL) rupture and reconstruction surgery (85). Lastly, we have completed a pilot study showing ALM therapy may have potential for treating burn trauma. We showed that small-volume ALM therapy protected the lung by significantly reducing oxidative stress (75% fall in malondialdehyde concentration), maintained alveolar and epithelial integrity, and improved cardiac function and O_2_ delivery in the first 8 h following a 30% total body surface area (TBSA) thermal burn in a rat model (86). Currently we are working on optimal dosages for ALM resuscitation and recovery after severe burns combined with hemorrhagic shock compared to standard-of-care Lactated Ringers. In summary, ALM may have clinical applications in a number of areas. While appreciating that the success rate of translating new drugs to humans is around 5% or less (87), the next phase is ALM drug development product formulation with all attending analytical methods for human application.

## Conclusion

ALM therapy is emerging as a systems-acting drug therapy that confers CNS-cardiovascular coupling with endothelial and mitochondrial protection after different trauma and infective states. The first pillar of ALM protection appears to be blunting the CNS stress response and a shift toward parasympathetic dominance, which has an overall effect to reduce secondary injury processes (coagulopathy, inflammation, immunosuppression, oxidative stress) and improve the transfer of O_2_ to tissue mitochondria. Future considerations include different roles of sex on ALM’s underling ability to treat traumatic injury and understanding how ALM ‘switches’ the system from an injury phenotype to a survival phenotype in different pathophysiological states. The amassed preclinical data in small and large animal models appears positive and supports future translation into humans after conducting the appropriate safety trials.

## Author contributions

GD, JM, and HL contributed equally to the design, implementation, literature analysis, and writing of the manuscript. All authors contributed to the article and approved the submitted version.
